# Supplementary material for: COVID-19 Vaccination Limits Systemic Danger Signals in SARS-CoV-2 Infected Patients
Source: Viruses. 2022 Mar 9;14(3):565. doi: 10.3390/v14030565 (PMC8949226; doi:10.3390/v14030565)
Supplement: Supplementary file 1 [file viruses-14-00565-s001.zip › viruses-1603038-supplementary.pdf]

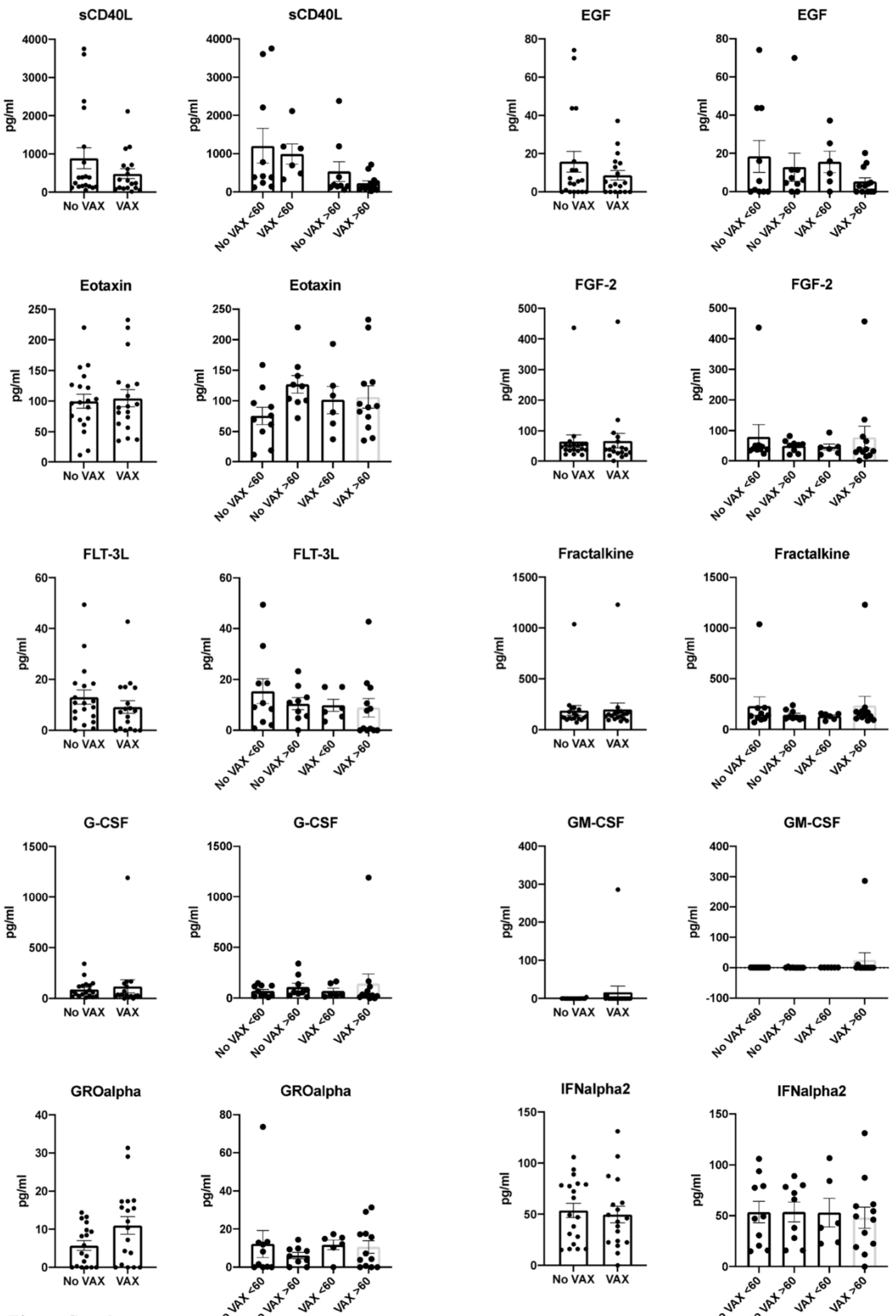

Figure Sup 1

Figure S1. circulating levels of the indicated analytes (18 cytokines) in NoVax and Vax patients.

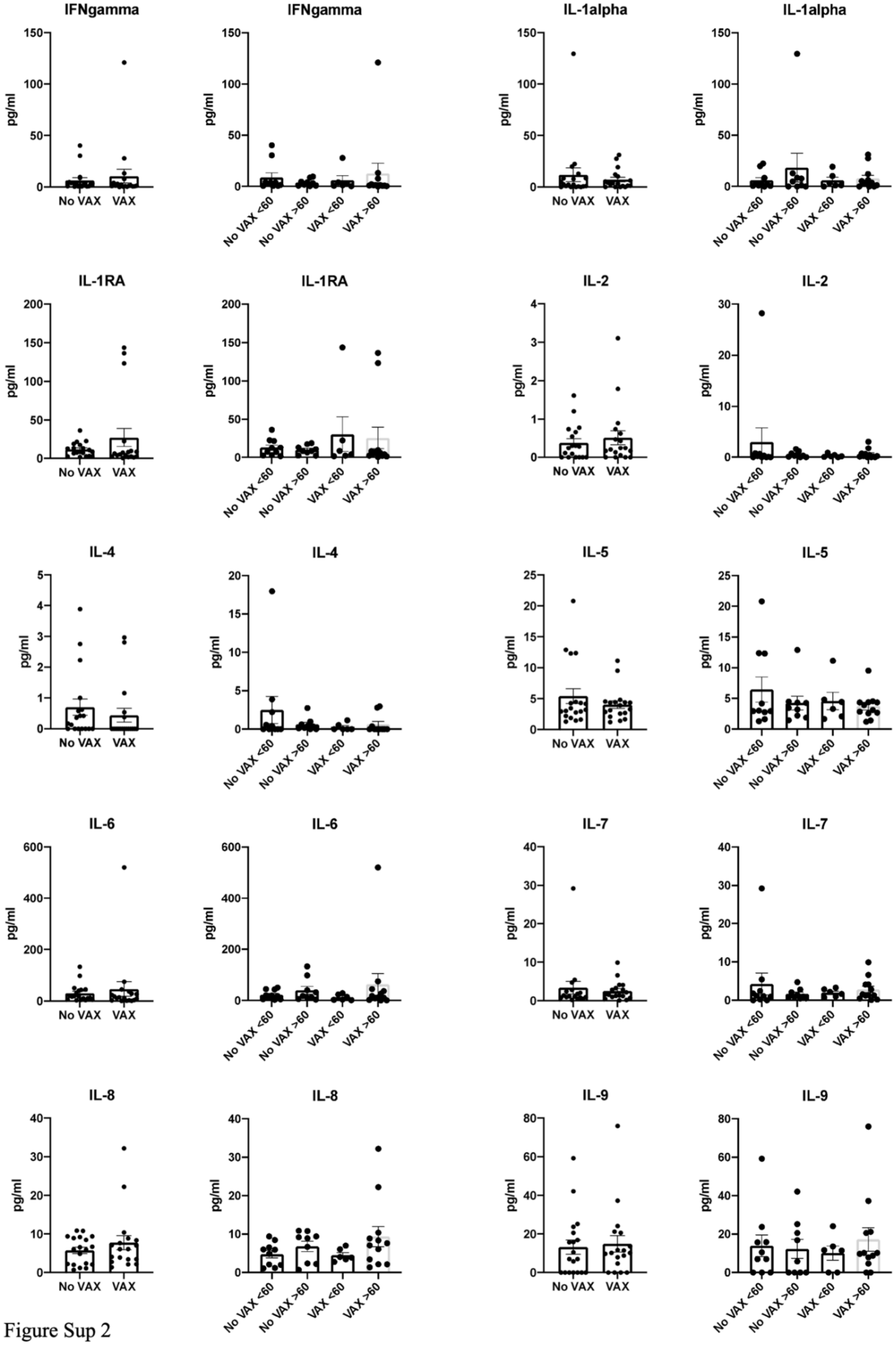

Figure Sup 2

Figure S2. circulating levels of the indicated analytes (18 cytokines) in NoVax and Vax patients.

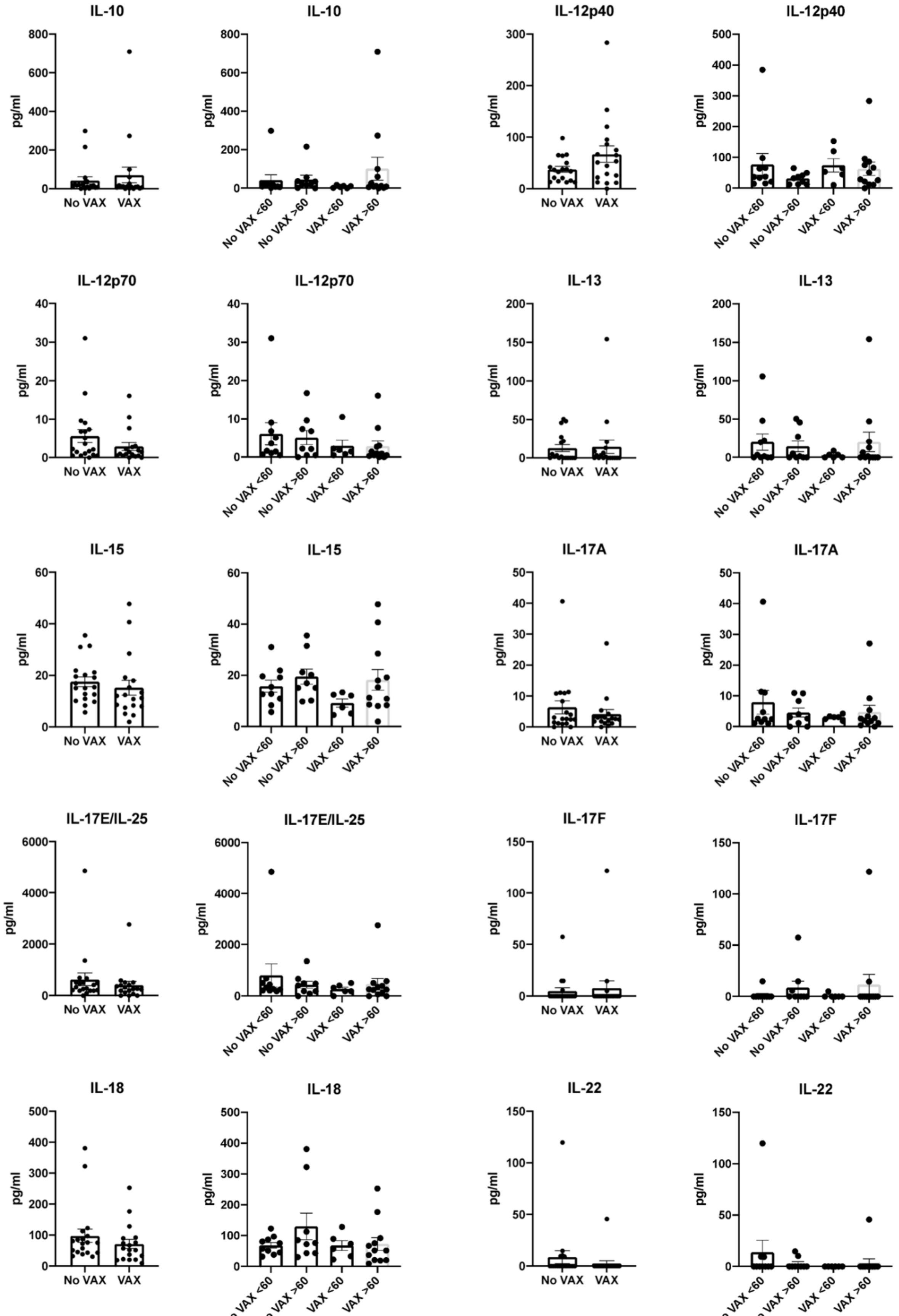

Figure Sup 3

Figure S3. circulating levels of the indicated analytes (10 cytokines) in NoVax and Vax patients.

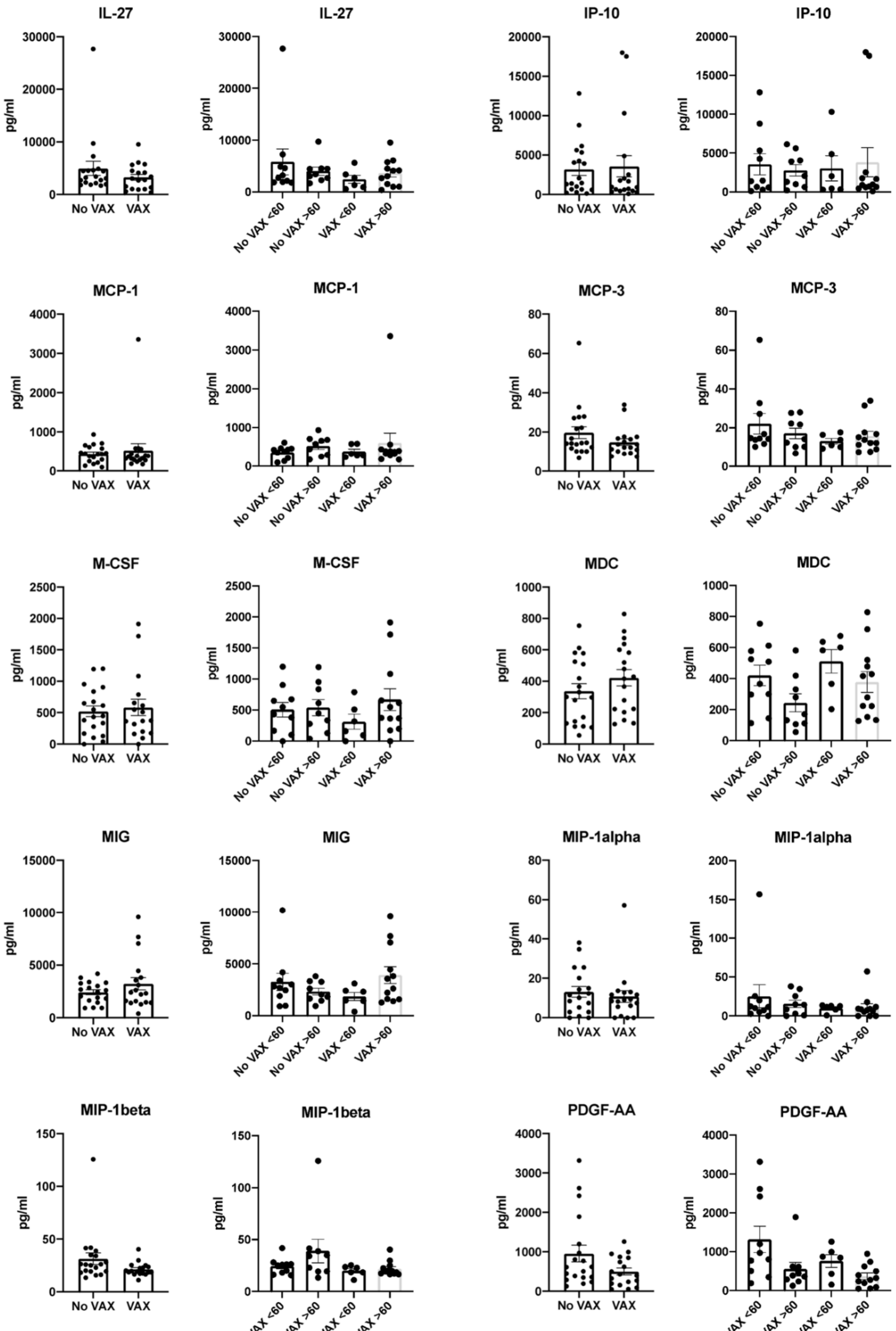

Figure Sup 4

Figure S4. circulating levels of the indicated analytes (10 cytokines) in NoVax and Vax patients.

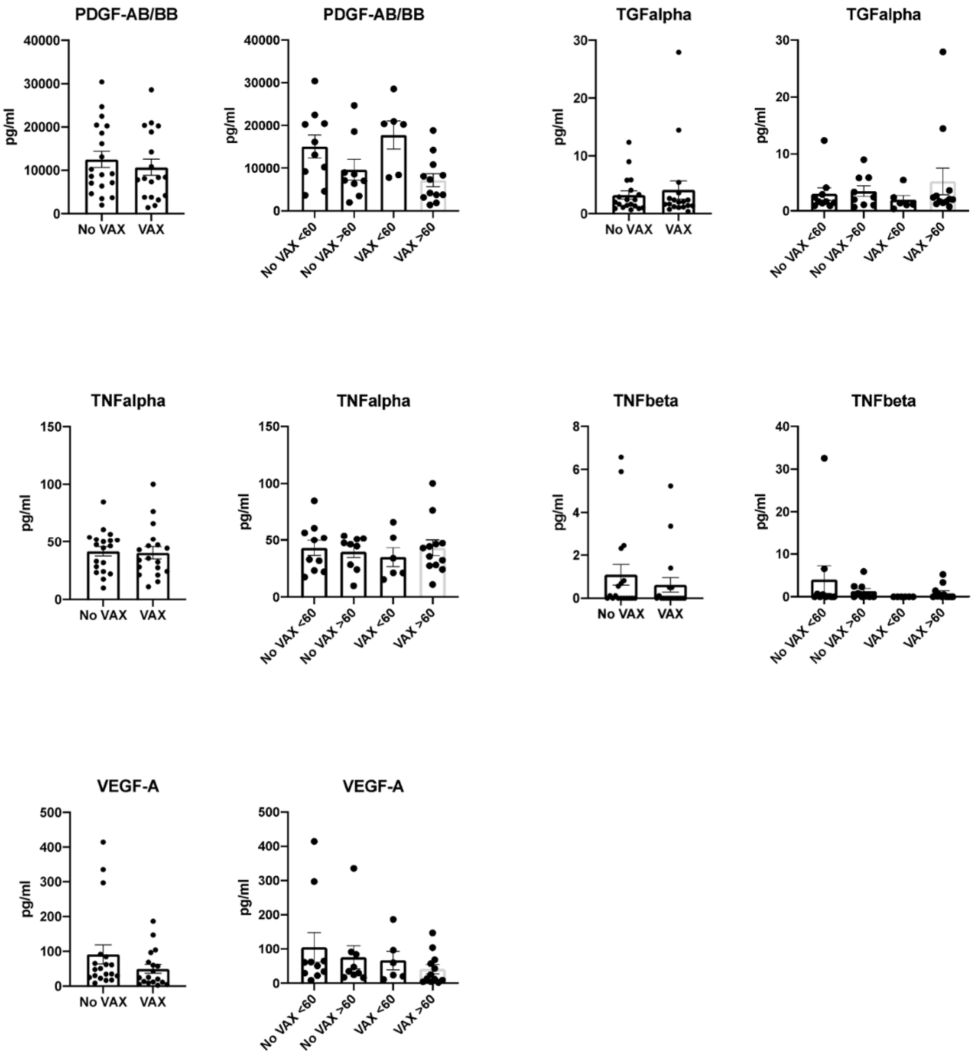

Figure S5. circulating levels of the indicated analytes (10 cytokines) in NoVax and Vax patients.

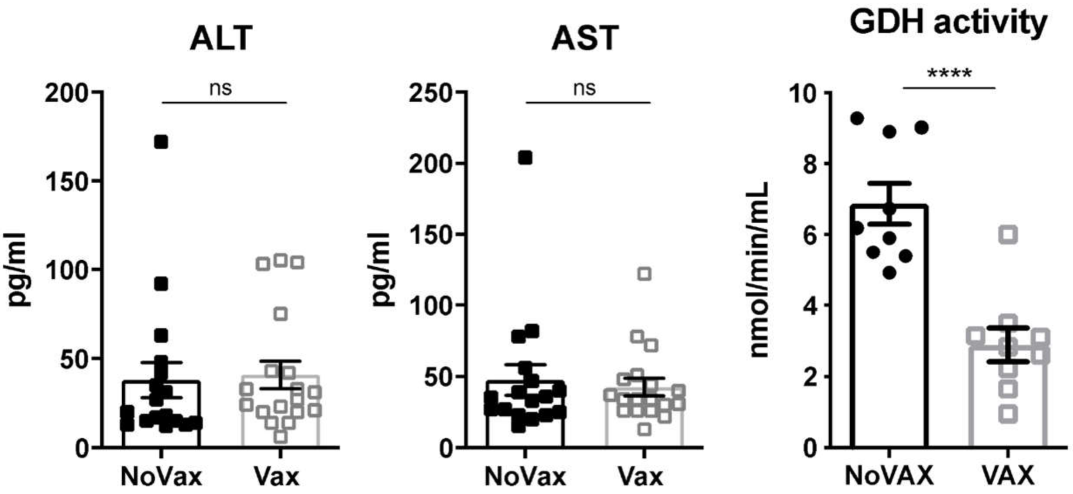

Figure S6. ALT and AST liver transaminases and GDH levels in NoVax and Vax patients. Differences were considered statistically significant at confidence levels *$P < 0.05$.
